# Modulation of Autophagy by a Small Molecule Inverse Agonist of ERRα Is Neuroprotective

**DOI:** 10.3389/fnmol.2018.00109

**Published:** 2018-04-09

**Authors:** S. N. Suresh, Aravinda K. Chavalmane, Malini Pillai, Veena Ammanathan, D. J. Vidyadhara, Haorei Yarreiphang, Shashank Rai, Abhik Paul, James P. Clement, Phalguni A. Alladi, Ravi Manjithaya

**Affiliations:** ^1^Molecular Biology and Genetics Unit, Jawaharlal Nehru Centre for Advanced Scientific Research, Bangalore, India; ^2^Department of Neurophysiology, National Institute of Mental Health and Neuro Sciences, Bangalore, India; ^3^Neuroscience Unit, Jawaharlal Nehru Centre for Advanced Scientific Research, Bangalore, India

**Keywords:** estrogen related receptor α, autophagy, XCT 790, small molecule screen, Parkinson’s disease, mTOR independent modulator, neuroprotection

## Abstract

Mechanistic insights into aggrephagy, a selective basal autophagy process to clear misfolded protein aggregates, are lacking. Here, we report and describe the role of Estrogen Related Receptor α (ERRα, HUGO Gene Nomenclature ESRRA), new molecular player of aggrephagy, in keeping autophagy flux in check by inhibiting autophagosome formation. A screen for small molecule modulators for aggrephagy identified ERRα inverse agonist XCT 790, that cleared α-synuclein aggregates in an autophagy dependent, but mammalian target of rapamycin (MTOR) independent manner. XCT 790 modulates autophagosome formation in an ERRα dependent manner as validated by siRNA mediated knockdown and over expression approaches. We show that, in a basal state, ERRα is localized on to the autophagosomes and upon autophagy induction by XCT 790, this localization is lost and is accompanied with an increase in autophagosome biogenesis. In a preclinical mouse model of Parkinson’s disease (PD), XCT 790 exerted neuroprotective effects in the dopaminergic neurons of nigra by inducing autophagy to clear toxic protein aggregates and, in addition, ameliorated motor co-ordination deficits. Using a chemical biology approach, we unrevealed the role of ERRα in regulating autophagy and can be therapeutic target for neurodegeneration.

## Introduction

Proteostasis machineries associated with the clearance of various cellular cargos including toxic proteins and damaged organelles in eukaryotic cells primarily include the chaperone, the Ubiquitin–Proteasome System (UPS), and the autophagy pathways (Hipp et al., 2014). UPS predominantly degrades short-lived proteins via tagging them with ubiquitin at specific amino acid residues (Hipp et al., 2014). The bulk degradation of long-lived proteins or organelles is mediated largely by the evolutionarily conserved cellular process referred to as macroautophagy (hereafter autophagy). A selective degradation mechanism called aggrephagy can help cells to clear the toxic, long-lived, aggregate-prone proteins. Misfolded and aggregate prone proteins are substrates for autophagy (Nixon, 2013). Intracellular accumulation of misfolded protein aggregates is an evident feature of several neurodegenerative diseases including Parkinson’s disease (PD). Owing to their hydrophobic nature, these aggregates sequester cellular proteins, thereby, perturbing cellular proteostasis machineries leading to neuronal death. Neurons are non-dividing cells and can’t dilute out the aggregates and, thus, more sensitive to proteotoxicity (Nixon, 2013). This condition is further exasperated upon aging as proteostasis efficiency decline. Recent studies highlight the importance of autophagy in curbing cellular cytotoxicity as a consequence of impaired clearance of aggregate prone proteins. Brain specific autophagy knockout mice (Atg5) display age related accumulation of protein aggregates, which eventually leads to neurodegenerative phenotypes, indicating that basal autophagy is vital for clearing protein aggregates (Hara et al., 2006). Additionally, tissue-specific knockdown of Atg7 in central nervous system of mice resulted in accumulation of inclusion bodies in autophagy-deficient neurons (Komatsu et al., 2006). Autophagy is shown to be dysfunctional during the neurodegenerative disease pathology (Nixon, 2013). Thus, restoration of autophagy through pharmacological approaches using small molecules has been reported to be neuroprotective (Sarkar et al., 2007; Khurana and Lindquist, 2010; Rajasekhar et al., 2015; Suresh et al., 2017). Small molecule that induces or restores defunct autophagy could aid in toxic aggregate clearance and essential for maintaining the cellular and organismal homeostasis (Rajasekhar et al., 2014, 2015). Broadly, small molecule autophagy modulators can be classified into mammalian target of rapamycin (MTOR)-dependent or -independent types, depending on its mechanism of action. Since MTOR has autophagy independent functions, targeting MTOR could have adverse side effects in patients with immunosuppression and impaired wound healing processes. Hence, this warrants for identifying new small molecules that are MTOR-independent with potent aggrephagy function/induction capabilities. More importantly, identifying the new molecular players that helps to decipher mechanistic interplay of autophagy and neuroprotective basic mechanisms remain a challenge.

In this study, we discovered a novel autophagy inducer, XCT 790, that was identified previously in our laboratory from a high-throughput screening of library containing pharmacologically active compounds (LOPAC^1280^) in yeast. XCT 790, a thiadiazoleacrylamide, is the most selective inverse agonist of the orphan nuclear receptor, Estrogen-Related Receptor α (ERRα; Busch et al., 2004) was identified as a “Hit”. Due to lack of any known natural ligand, XCT 790 has been used as a tool to delineate the lesser known functions of ERRα in different biological processes (Ariazi and Jordan, 2006). XCT 790 cleared α-synuclein aggregates in an autophagy-dependent manner in human neuronal cells. It significantly induced autophagy through an MTOR-independent mechanism and ERRα-dependent manner. This neuroprotective compound uncovers the role of ERRα in a basic autophagy pathway. We found that ERRα inhibits autophagy in fed conditions, thus, helps in regulating the basal autophagic flux. Additionally, in a preclinical mouse model of PD, XCT 790 was found to have a neuroprotective role through clearing the toxic protein aggregates as evidenced by immunohistological and behavior analyses.

## Materials and Methods

### Chemicals and Antibodies

XCT 790 (X4753), 1-methyl-4-phenyl-1,2,3,6-tetrahydropyridine (MPTP, M0896), anti LC3 antibody (L7543), anti-FLAG antibody (F3165), 3-Methyl Adenine (3-MA) (M9281), DMEM F-12 (D8900), Penicillin and Streptomycin (P4333), DMEM (D5648), 3,3′-Diaminobenzidine (DAB, D3939), Trypsin EDTA (59418C), and Atto 663 (41176) were purchased from Sigma-Aldrich. Anti-phospho 4E-BP1T37/46 antibody (2855) and total 4E-BP1 antibody (9452), Anti-phospho P70S6K T389 antibody (9239) and total P70S6K antibody (9202), Anti-phospho AMPK antibody (8359) and total AMPK antibody (8359), Anti-phospho ULK1 antibody (8359) and total ULK1 antibody (8359) and anti-rabbit IgG, HRP (7074) antibody were purchased from Cell Signaling Technology. Cell Titre Glo^®^ kit (G 7571) was procured from Promega. Anti-GAPDH (MA5-15738) and anti-β-tubulin (MA5-16308) antibodies were purchased from Thermo Scientific. Anti-p62 (ab 56416), Anti-ERRα (ab 16363) and Anti-Pgk1 (ab 38007) antibody were purchased from Abcam. Anti-EGFP (11 814 460 001) antibody was purchased from Roche. Anti-mouse IgG, and HRP (172-1011) antibody were purchased from Bio-Rad. Anti-A11 (AB9234) was purchased from Merck Millipore. Anti-Tyrosine hydroxylase (TH; N196) antibody was purchased from Santa Cruz Biotechnology. FITC conjugated anti-rabbit secondary antibody (F7512), and Cy3 conjugated anti-rabbit secondary antibody (C2306) were purchased from Sigma-Aldrich. CMAC-Blue (C2110) was purchased from Life Technologies. Bafilomycin A1 (11038) was purchased from Cayman chemical. VECTASTAIN Elite ABC Kit (PK-6101) was purchased from VECTOR laboratories.

### siRNA, Plasmid Constructs and Bacterial Strains

For mammalian cell culture studies, plasmids used were ptf LC3 (Kimura et al., 2007; gift from Tamotsu Yoshimori, Addgene #21,074), EGFP-synuclein (Furlong et al., 2000; gift from David Rubinzstein, Addgene number #40,822). For infection studies, strains used were untagged or mCherry plasmid (Addgene #36,084) expressing *S. typhimurium* SL1344 (gift from Prof. C. V. Srikanth, RCB, India). pCMV flag ERRα was a gift from Toren Finkel (Ichida et al., 2002; Addgene plasmid #10,975). ERRα siRNA (L-0, 03, 403–00) and scrambled siRNA (D-001810-10-05) were procured from Dharmacon.

### Mammalian Cell Culture, Infection and Autophagy Assays

**Figure 1:**
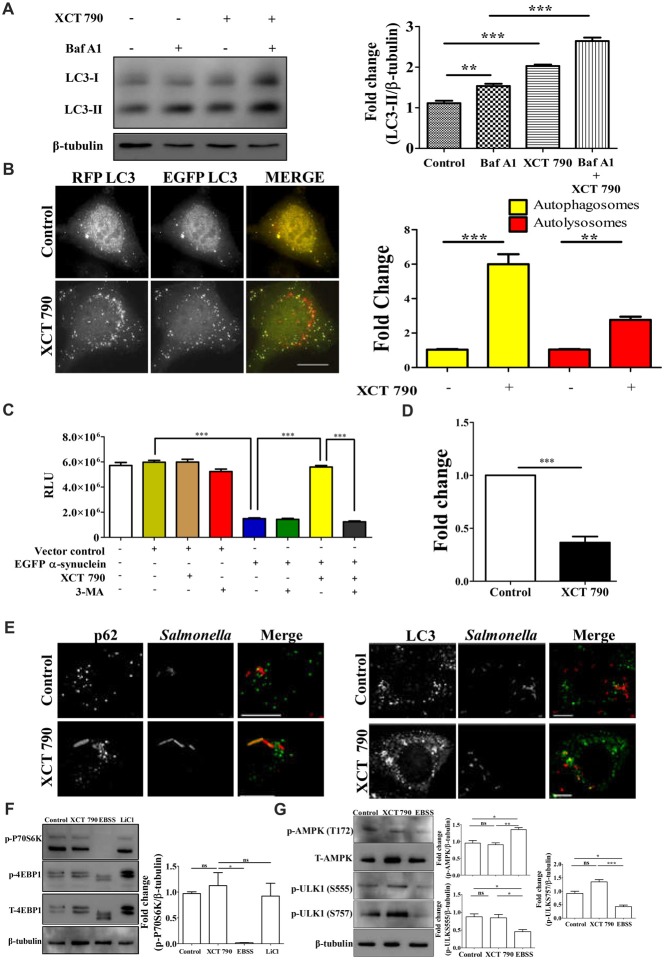
XCT 790 modulates aggrephagy and xenophagy in mammalian cells. **(A)** Representative Western blot of LC3 processing assay in SH-SY5Y cells treated with XCT 790 (2 h) under growth condition and normalized LC3-II levels were quantified (*n* = 3). β-tubulin was used as a loading control. Statistical analysis was performed using one-way ANOVA and *post hoc* Bonferroni test. Error bars, mean ± SEM. ns-non significant, **P < 0.01, ***P < 0.001. **(B)** Representative microscopy images of tandem RFP-EGFP-LC3 assay in HeLa cells treated with XCT 790 for 2 h. Yellow puncta was autophagosomes and red was autolysosomes. Fold change in autophagosomes and autolysosomes by XCT 790 were quantified (*n* = 50 cells and three independent experiments). Scale bar was 15 μm. Statistical analysis was performed using one-way ANOVA and *post hoc* Bonferroni test. Error bars, mean ± SEM. ns-non significant, **P < 0.01, ***P < 0.001. **(C)** Graph indicating the cell viability read out of SH-SY5Y overexpressing EGFP-α-synuclein treated with XCT 790 in presence of pharmacological autophagy inhibitor 3-MA. Cell viability was analyzed using CellTitre Glo (Promega) assay. More RLU readout was indicative of more cell viability and vice-versa (three independent experiments). Statistical analysis was performed using one-way ANOVA and *post hoc* Bonferroni test. Error bars, mean ± SEM. ***P < 0.001. **(D)** Graph for colony forming unit (CFU)s indicating the intracellular burden of *S. typhimurium* treated with XCT 790 (10 μM) for 6 h. CFU represent survival of *S. typhimurium* within the host cells. Fold change between the untreated and XCT 790 treated samples were quantified (three independent experiments). Statistical analysis was performed using two-tailed paired *t*-test. Error bars, mean ± SEM. ns-non significant, **P < 0.01, ***P < 0.001. **(E)** Representative microscopy images of HeLa cells infected with mCherry expressing *S. typhimurium* treated with XCT 790 for 6 h. Cells were immunostained for either p62 or LC3 in untreated and XCT 790 treated samples (*n* = 25 cells and three independent experiments). Scale bar 10 μm. **(F)** Representative Western blots of MTOR substrates—P70S6K (phospho and total form) and 4EBP1 (phospho and total form)—regulation by various treatments like XCT 790, EBSS and LiCl. β-tubulin was used as a loading control. Normalized p-P70S6K levels were quantified for three independent experiments. Statistical analysis was performed using one-way ANOVA and the *post hoc* Bonferroni test. Error bars, mean ± SEM. ns-non significant, *P < 0.05. **(G)** Representative Western blots of signaling pathway proteins like AMPK (phospho and total form) and ULK1 (phospho and total form) regulation by XCT 790 and EBSS. Normalized p-AMPK, p-ULK1 (S555), p-ULK1 (757) levels were quantified for three independent experiments. β-tubulin was used as a loading control. Statistical analysis was performed using one-way ANOVA and the *post hoc* Bonferroni test. Error bars, mean ± SEM. ns-non significant, *P < 0.05, **P < 0.01, ***P < 0.001.β-tubulin was used as a loading control. Concentrations of XCT 790, 3-MA and LiCl used were 5 μM, 100 nM and 10 mM.

SH-SY5Y cells were cultured in DMEM-F12 containing 10% FBS (Life technologies). HeLa cells were cultured in DMEM containing 10% FBS (Pan-Biotech). Cell lines were maintained at 37°C and 5% CO_2_. The autophagy assays were performed by seeding equal numbers of sub-confluent HeLa or SH-SY5Y cells in 6-well dishes and allowed to attach for 24 h, then treated with XCT 790 (5 μM, Figure 1, Supplementary Figures S1A,B) and/or 3-MA (5 mM) and/or lithium chloride (10 mM) in fed condition for 2 h. After treatments, the cell lysates were analyzed by immunoblotting.

RFP-EGFP-LC3 assay: sub-confluent HeLa and/or SH-SY5Y cells were seeded into 60 mm cell culture dishes, then transfected with ptf LC3 construct and/or siRNA, and allowed to express for 48 h. Cells were trypsinized, seeded again on poly-D-lysine coated cover slips in a 12 or 24 well plates and allowed to attach. After appropriate treatments, the coverslips containing cells were processed for imaging. For immunofluorescent antibody staining, the cover slips were incubated in primary antibody at 4°C for overnight followed by secondary antibody incubation at room temperature.

Intracellular colony forming unit (CFU) assay: *S. typhimurium* SL1344 were grown overnight at 37°C under micro-aerophilic conditions. HeLa cells were infected at a multiplicity of infection (MOI) of 200 for 1 h. The cells were treated with media containing gentamycin (100 μg/ml) for 1 h to kill the extracellular bacteria. The cells were then treated with XCT 790 (10 μM) and incubated further for 4 h. The HeLa cells were lysed using lysis buffer (0.1% SDS, 1% Triton X-100, 1× PBS) and the intracellular *Salmonella* were plated on LB plates, incubated overnight at 37°C, and the CFU was counted.

### Immunoblot Analysis

Mammalian cell lysates preparation: after treatments, cells were collected in Laemmli buffer to perform LC3 processing assay, P70S6K, AMPK, ULK1 and 4E-BP1 immunoblotting. Samples were electrophoresed onto SDS-PAGE (8%–15%) and then transferred onto PVDF (Bio-Rad) membrane through Transblot turbo (Bio-Rad). Blots were stained with Ponceau S, and then probed with appropriate primary antibodies at 4°C for overnight and subsequently HRP-conjugated secondary antibody. Signals were attained using enhanced chemiluminescence substrate (Clarity, Bio-Rad) and imaged using a gel documentation system (G-Box, Syngene) and then bands were quantitated using ImageJ software (NIH).

### Microscopy

For imaging the mammalian cells, after appropriate treatments, coverslips containing cells were fixed using 4% paraformaldehyde (PFA; Sigma) and then permeabilized using Triton X-100 (0.2%, HiMedia). Coverslips were mounted on slide using antifade, Vectashield mounting medium (Vector Laboratories). For antibody staining, coverslips were blocked using 5% BSA for 1 h at room temperature, then incubated with primary antibody at 4°C, overnight and then subsequently probed with corresponding fluorescent dye conjugated secondary antibody.

Images were acquired using DeltaVision Elite widefield microscope (API, GE) with following filters: FITC (490/20 and 529/38), TRITC (542/27 and 594/45) and Cy5 (632/22 and 676/34). Acquired images were processed using DV softWoRX software.

### Cell Viability Assay

SH-SY5Y cells were seeded onto tissue culture treated 96-well plate and then transfected with EGFP-α-synuclein only, and/or co-transfected with siRNA. To cells, appropriate drugs were added (24 h) after 48 h of transfection. Using luminescence-based CellTitre-Glo^®^ (Promega) kit, the cell viability was assayed using automated microtitre plate reader Varioskan Flash (Thermo Scientific).

### Animal Studies

All procedures in this study were approved by Committee for the Purpose of Control and Supervision of Experiments on Animals (CPCSEA) and JNCASR Institutional Animal Ethics Committee and conducted as per their guidelines. Inbred male C57BL/6J mice (3–4 months old) were used for all experimental groups (*n* = 6). The animals were maintained under standard laboratory conditions i.e., temperature 25° ± 2°C, 12 h light: 12 h dark cycle and 50 ± 5% relative humidity with *ad libitum* access to food and water.

### MPTP.HCl and XCT 790 Treatment

The mice were distributed into three groups: vehicle, MPTP and MPTP+XCT 790, and injections were administered intraperitoneally. The vehicle group was injected with dimethyl sulfoxide (DMSO) i.e., the solvent. The MPTP group received 23.4 mg/kg MPTP.HCl in 10 ml/kg body weight of saline, administered four times at 2 h interval (Jackson-Lewis and Przedborski, 2007). The MPTP+XCT 790 group mice were injected with 5 mg/kg body weight of XCT 790 dissolved in DMSO, alongside the first MPTP injection. The treatment was continued by administering XCT 790 in “an injection a day regime” for 6 days. All the mice were sacrificed 7 days after MPTP administration and the brains were processed for immunohistochemistry.

### Tissue Processing for Immunohistochemistry

The mice were anesthetized using halothane inhalation and perfused intracardially with saline, followed by 4% buffered PFA (pH 7.4). The brains were removed quickly and post fixed in the same buffer for 24 h to 48 h at 4°C and cryoprotected in an increasing gradient of sucrose. Coronal midbrain cryosections of 40 μm thick were collected serially on gelatinized slides. Every sixth midbrain section was used for immunostaining.

### Immunoperoxidase Staining of Tyrosine Hydroxylase (TH)

The immunoperoxidase labeling protocol was a slight modification of that reported earlier (Vidyadhara et al., 2016). Briefly, the endogenous expression of peroxidase was quenched using 0.1% H_2_O_2_ in 70% methanol, followed by blocking of non-specific staining by 3% buffered solution of bovine serum albumin for 4 h at room temperature. The sections were then incubated with the rabbit polyclonal anti-TH antibody (1:800, Santa Cruz Biotechnology Inc., Santa Cruz, CA, USA), followed by anti-rabbit secondary antibody (1:200 dilution; Vector Laboratories, Burlingame, CA, USA). The tertiary labeling was performed using avidin–biotin complex solution (1:100, Elite ABC kits; Vector Laboratories, Burlingame, CA, USA). The staining was visualized using 0.05% solution of DAB, in 0.1 M acetate imidazole buffer (pH 7.4) with 0.1% H_2_O_2_. Phosphate buffered saline (0.01 M) containing 0.3% Triton X-100 (0.01 M PBST, pH 7.4) was used as both diluent and washing buffer. Appropriate negative controls were processed identically.

### Stereological Quantification of TH-Immunoreactive (TH-ir) Neurons at SNpc

Stereological quantification of TH-ir dopaminergic neurons was performed using optical fractionator probe (Vidyadhara et al., 2016, 2017). The SNpc was delineated on every sixth TH-ir midbrain section (Fu et al., 2012) using 4× objective of the Olympus BX61 Microscope (Olympus Microscopes, Japan) equipped with StereoInvestigator (Software Version 7.2, Micro-Brightfield Inc., Colchester, VT, USA). The cells were counted using oil immersion lens (100×), with a regular grid interval of 22,500 μm^2^ (*x* = 150 μm, *y* = 150 μm) and counting frame of 3600 μm^2^ (*x* = 60 μm, *y* = 60 μm). The mounted thickness averaged to 25 μm. A guard zone of 4 μm was implied on either side, thus, providing 17 μm of z-dimension to the optical dissector. The quantification was performed starting with the first anterior appearance of TH-ir neurons in SNpc to the caudal most part in both hemispheres and added to arrive at the total number. The volume of SNpc was estimated by planimetry.

### Densitometry Based Image Analysis

The offline evaluation of TH expression was performed on high magnification images of TH immunostained nigral dopaminergic neurons using Q Win V3 (Leica Systems, Germany); a “Windows” based image analysis system (Alladi et al., 2010; Vidyadhara et al., 2017). A cumulative mean was derived from the values obtained from sampling approximately 200 dopaminergic neurons per animal and expressed as gray values on a scale of 0–255, where “255” meant absence of staining and “0” equaled intense staining.

### Immunofluorescence Based Double Staining of SNpc Dopaminergic Neurons

The sequential immunolabeling procedure was used to co-label the TH and LC3 and/or A11 (Alladi et al., 2010). First, the midbrain sections were equilibrated with 0.1 M PBS (pH 7.4) for 10 min and then incubated with buffered bovine serum albumin (3%) for 4 h to block non-specific epitopes. Then, the sections were incubated in rabbit anti-LC3 antibody (1:1000) and/or anti-oligomer antibody (A11, 1:1000) for 72 h at 4°C. After subsequent washes, the sections were incubated in corresponding fluorescent secondary antibody (1:200) overnight at 4°C. Co-labeling with TH was performed on the same sections using rabbit anti-TH antibody (1:500), followed by secondary labeling. PBST (0.01 M, pH 7.4) was used as both working and washing buffer. The sections were then mounted using Vectashield mounting medium.

### Behavioral Studies

Open Field and Rotarod experiments were performed using 3–4-month-old C57BL/6J male mice. The experimental procedures were modified from references: Brooks and Dunnett (2009), Patil et al. (2014) and Liu et al. (2015). All the animals were handled for three consecutive days by experimenters prior to the start of training session. Mice were habituated to behavior room (light intensity maintained at 100 lux) for 15 min before the commencement of handling, training and tests. The health of each mouse was monitored every day before training or test sessions by recording their body weight. The behavioral experiments were scheduled in such a way that the fatigue of one behavioral test does not affect the other. Thus, Open Field trials (low stress activity) were done in forenoon, whereas, Rotarod trials (high stress activity) were performed in the afternoon. Double blind approach was taken in which the experimenters were unaware of the drug treatment given to the animals as well as the results of data analysis. Data were represented as bar diagram using GraphPad prism 5 software.

### Open Field Trials

Open Field tests were done in a wooden box (Open Field arena) of dimensions: 50 cm × 50 cm × 45 cm in which the internal surface was coated with white polish. The Open Field arena was custom made in JNCASR. Mice were trained in an Open Field arena for two consecutive days prior to the day of actual tests. During training or tests, a single mouse was left in the Open Field arena for 5-min to explore. The activity of mouse was recorded with the help of a digital camera (SONY^®^ color video camera, Model no. SSC-G118) supported by the software SMART v3.0.04 (Panlab Harvard Apparatus, Holliston, MA, USA). At the end of 5-min, the mouse was removed and returned to its home cage. The Open Field arena was cleaned with 70% ethanol and dried before placing the next mouse in it. The distance traveled in the zone periphery was calculated by SMART v3.0.04 software (Panlab Harvard Apparatus, Holliston, MA, USA). The data were compared, analyzed and represented as bar diagrams by using the software GraphPad prism5.

### Rotarod Trials

Rotarod was custom made at the mechanical workshop, National Centre for Biological Sciences, Bengaluru, India. The instrument includes a textured horizontal rod (diameter 3.3 cm) made of Delrin. The rod was fixed at a height of 30 cm above a platform, which was cushioned for comfortable fall of mice from rotating rod. The rod was divided into three small areas (9.3 cm each) by fitting two circular discs (diameter 40 cm) made up of Teflon to allow three mice, completely shielded from each other, to be trained simultaneously. The speed of rotation was incremented from 5 rpm to 20 rpm manually by an electric motor fitted in the instrument. The mice were trained for five consecutive days prior to the day of injection. The training was given by gradually increasing the speed of the rod from lower to higher rpm by accelerating at the rate of 1 rpm/5 s. On 1st day, the mice were trained at 5–10 rpm, on 2nd day 7–12 rpm, on 3rd day 13–20 rpm and at 20 rpm (fixed) on 4th and 5th day. During training and tests, mice were placed on the non-rotating rod and were allowed to balance themselves before increasing the speed manually. For every rpm, the mice were given a stabilization period of 5 s. Mice that failed to learn to stabilize on the rod were given two more chances. Once the stabilization period was completed successfully, the mice were allowed to run on the rotating rod for 60 s at the respective rpm. The entire Rotarod trial was recorded on a digital camera (SONY^®^ HDR-CX405) and the latency to fall was calculated manually. The data obtained were compared, analyzed and plotted as mean latency to fall in the form of bar diagrams using GraphPad prism5.

### Statistical Analysis

One-way or Two-way ANOVA followed by Bonferroni’s *post hoc* test was applied to derive statistical significance. Values were expressed as mean ± SEM.

## Results

### Modulation of Mammalian Aggrephagy and Xenophagy by XCT 790

Toxic protein aggregates and intracellular pathogens are known to be substrates of the autophagy pathway for their effective cellular degradation (Deretic and Levine, 2009; Nixon, 2013). Based on a previous small molecule screening for aggrephagy inducers from our laboratory (Suresh et al., 2017), we identified thiadiazoleacrylamide, XCT 790, as a drug-like molecule that abrogates α-synuclein cellular toxicity, and intracellular *Salmonella typhimurium*. We tested the potential of XCT 790 to clear toxic α-synuclein protein aggregates, and *Salmonella* burden through autophagy in mammalian cells such as human neuroblastoma SH-SY5Y and HeLa cell lines.

To test the modulation of mammalian autophagy and its flux by XCT 790, we used immunoblot analysis based LC3 (autophagosome marker) and microscopy-based tandem RFP-EGFP-LC3 assays. In tandem RFP-EGFP-LC3 assay, XCT 790 treatment significantly induced autophagosomes and autolysosomes formation in both SH-SY5Y (control vs. XCT 790 treated, autophagosomes, ~2-fold, *P* < 0.05; autolysosomes, ~4-fold, *P* < 0.01 Supplementary Figure S2A) and HeLa cells (control vs. XCT 790 treated, autophagosomes, ~5-fold, *P* < 0.001; autolysosomes, ~2-fold, *P* < 0.001 Figure 1B). Additionally, XCT 790 treatment enhanced accumulation of LC3-II levels indicating the induction of autophagy (~2.5-fold, untreated vs. XCT 790, *P* < 0.001, Figure 1A). These results clearly demonstrated that XCT 790 modulates mammalian autophagy.

We then addressed whether XCT 790 protects SH-SY5Y cells from EGFP-α-synuclein mediated toxicity. Overexpression of EGFP-α-synuclein in SH-SY5Y cells was toxic leading to significant cell death as measured by cell viability assay (~4-fold, vector control or untransfected vs. α-syn transfected, Figure 1C). Upon administration of XCT 790 to cells overexpressing EGFP-α-synuclein, the cell viability increased significantly than that of untreated cells (~4-fold, α-syn over expressed cells, untreated vs. XCT 790 treated, *P* < 0.001, Figure 1C) and comparable to that of vector control (vector control vs. α-syn over expressed cells XCT 790 treated, ns, *P* > 0.05, Figure 1C). We observed that the potential of XCT 790 to protect cells from EGFP-α-synuclein toxicity is abrogated in presence of pharmacological autophagy inhibitor, 3-MA (α-syn over expressed cells, XCT 790 vs. XCT 790 + 3-MA, ~4-fold, *P* < 0.001, Figure 1C) that is comparable to that of XCT 790 untreated cells (α-syn over expressed cells, untreated vs. XCT 790 + 3-MA, ns, *P* > 0.05, Figure 1C). These results clearly demonstrate that XCT 790 protects human neuroblastoma cells from EGFP-α-synuclein mediated toxicity in an autophagy dependent manner.

We also checked if XCT 790 clears the intracellular bacterial cargo. Upon XCT 790 treatment, we observed significant reduction in the intracellular *S. typhimurium* SL1344 compared to untreated (~2-fold, untreated vs. XCT 790 treated, *P* < 0.001, Figure 1D). We also observed increased recruitment of autophagy adaptor protein p62 and autophagosome membrane marker LC3 to *S. typhimurium* SL1344 (Figure 1E).

This study identifies XCT 790 as an autophagy inducer with a potential to clear toxic protein aggregates. We demonstrate that XCT 790 exerts protection to the cells against EGFP-α-synuclein mediated toxicity by inducing autophagy which helps clear the toxic aggregates.

### XCT 790 Modulates Autophagy Through an MTOR Independent Pathway

Autophagy is regulated by MTOR-dependent and MTOR-independent pathways that are amenable to chemical perturbations (Kim et al., 2011). To delineate the mechanism of autophagy modulation by XCT 790, we examined the activity of MTOR through monitoring its substrates such as P70S6K and 4EBP1. Upon XCT 790 treatment, MTOR activity was unaffected as revealed by its substrates such as phospho-P70S6K and phospho-4EBP1 protein levels which were comparable to that of nutrient rich condition (Figure 1F; Supplementary Figure S2B). In contrast, the levels of phospho-P70S6K and phospho-4EBP1 were attenuated under starvation conditions where autophagy was regulated in an MTOR-dependent manner. Lithium Chloride (10 mM) is known to induce autophagy through an MTOR independent mechanism served as a positive control (Sarkar et al., 2005; Figure 1F, Supplementary Figure S2B). These observations asserted that XCT 790 is MTOR independent autophagy modulator.

We further examined whether XCT 790 exerts its effect through AMPK pathway, one of the predominant MTOR-independent mechanisms known to regulate autophagy. It was observed that treatment of XCT 790 for 2 h did not affect the activity of AMPK, as evident by the unchanged T172 phosphorylation of AMPK (Figure 1G) when compared to nutrient rich conditions. AMPK promotes autophagy in an MTOR-independent manner by directly activating ULK1 through phosphorylation of Ser 555 (S555). Whereas, under nutrient sufficiency, high MTOR activity inhibits ULK1 activation by phosphorylating ULK1 at Ser 757 (S757) and disrupting the interaction between ULK1 and AMPK (Kim et al., 2011). Therefore, we further examined the regulation of levels of activating (S555) and inhibitory (S757) phosphorylation of ULK1 by XCT 790. Consistent with unchanged levels of phosphorylated AMPK after treatment with XCT 790 for 2 h, the downstream phosphorylation of ULK1 at S555 was unaffected and comparable to the nutrient rich conditions (Figure 1G). This suggests that XCT 790 does not exert its effects through AMPK pathway. Importantly, MTOR-dependent phosphorylation of ULK1 at S757 remained unaltered in XCT 790-treated cells unlike in starvation conditions, where a concomitant decrease in the phospho-ULK1 S757 protein levels is observed. These results further confirm that XCT 790 acts through an MTOR-independent mechanism but not through AMPK pathway.

### XCT 790 Induces Autophagy Through Regulation of Estrogen-Related Receptor Alpha (ERRα)

XCT 790 was found to be the first potent and selective inverse agonist of ERRα (Busch et al., 2004). To elucidate the role of ERRα in contributing to the function of XCT 790 as autophagy inducer, we used the following two approaches: (a) siRNA-based silencing of ERRα; and (b) over expression of ERRα.

**Figure 2:**
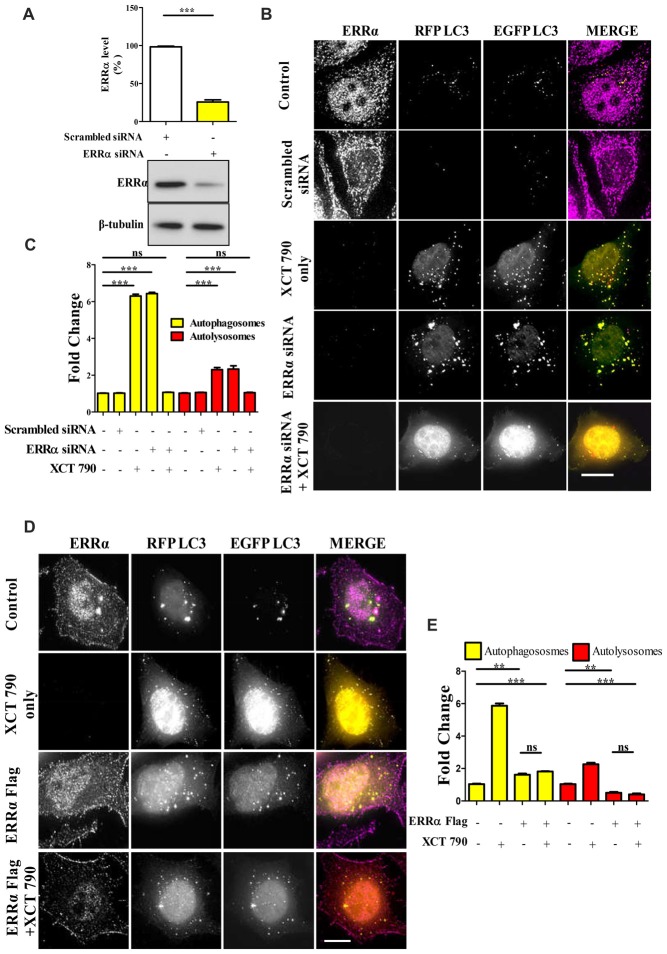
XCT 790 modulates autophagy through estrogen related receptor α (ERRα). **(A)** ERRα protein levels after transfecting either scrambled siRNA (100 picomoles) or ERRα siRNA (100 picomoles) for 48 h in HeLa cells was analyzed by Western blotting and then quantified (*n* = 3). β-tubulin was used as a loading control. Statistical analysis was performed using two-tailed paired *t*-test. Error bars, mean ± SEM. ***P < 0.001. **(B,C)** Microscopy images **(B)** of tandem RFP-EGFP-LC3 assay in XCT 790 treated HeLa cells (2 h) post ERRα siRNA transfection (48 h). Cells were immunostained for ERRα in various treatments. Scale bar was 15 μm. Quantification **(C)** of autophagosomes (yellow puncta) and autolysosomes (red puncta) modulated by XCT 790 treatment in ERRα siRNA transfected cells (*n* = 50 cells and three independent experiments). Statistical analysis was performed using one-way ANOVA and the *post hoc* Bonferroni test. Error bars, mean ± SEM. ns-non significant, ***P < 0.001. **(D,E)** Microscopy images **(D)** of tandem RFP-EGFP-LC3 assay in XCT 790 treated HeLa cells (2 h) post ERRα Flag transfection (48 h). Cells were immunostained for ERRα in all treatment groups. Scale bar used was 15 μm. Quantification **(E)** of autophagosomes (yellow puncta) and autolysosomes (red puncta) modulated by XCT 790 treatment in ERRα Flag transfected cells (*n* = 50 cells and three independent experiments). Statistical analysis was performed using one-way ANOVA and the *post hoc* Bonferroni test. Error bars, mean ± SEM. ns-non significant, **P < 0.01, ***P < 0.001. Concentration of XCT 790 used was 5 μM.

To evaluate the level at which the knockdown exerted its effect, cells were transfected with siRNAs targeting ERRα. A non-targeting pool was used as a control. The effects of knockdown on regulation of autophagy by ERRα 48 h post-transfection were monitored by microscopy-based tandem RFP-EGFP-LC3 assays. Knockdown efficiency was confirmed by Western blotting to be around 80% (Scrambled vs. ERRα siRNA, *P* < 0.001, Figure 2A). Consistent with the effect of XCT 790, knockdown of ERRα also resulted in a significant induction of autophagosomes (~5-fold, Scrambled vs. ERRα siRNA treated, *P* < 0.001, Figure 2B) and autolysosomes (~3-fold, Scrambled vs. ERRα siRNA treated, *P* < 0.001, Figure 2B). Autophagosome and autolysosome numbers in XCT 790 treated and ERRα downregulated cells were found to be comparable. These results suggested that XCT 790 modulated autophagy through ERRα.

We addressed this question through another approach to understand the autophagy modulation upon overexpression of ERRα. In ERRα over expressed cells, we found more autophagosomes (~2-fold, *P* < 0.01, ERRα overexpressed vs. untreated) and less autolysosomes (~2-fold, *P* < 0.01, ERRα overexpressed vs. untreated) than that of control (Figures 2D,E). From this, we could interpret that autophagy was inhibited at its autophagosome to lysosome fusion step upon over expression of ERRα. When XCT 790 was treated in ERRα over expressed cells, more autophagosomes (~2-fold, *P* < 0.01, ERRα overexpressed vs. untreated) and less autolysosomes (~2-fold, *P* < 0.01, ERRα overexpressed vs. untreated) than that of untreated were found (Figures 2D,E). This autophagic scenario was similar to that of only ERRα over expressed cells (Autophagosomes; ERRα over expressed + XCT 790 vs. ERRα overexpressed only, ns, *P* > 0.05 and Autolysosomes; ERRα over expressed + XCT 790 vs. ERRα overexpressed only, ns, *P* > 0.05, Figures 2D,E). When ERRα was over expressed, the autophagic modulating ability of XCT 790 was indeed abrogated.

Collectively, these results suggest that XCT 790 modulates autophagy through ERRα.

### ERRα Regulates Autophagy by Localizing Onto Autophagosomes

**Figure 3:**
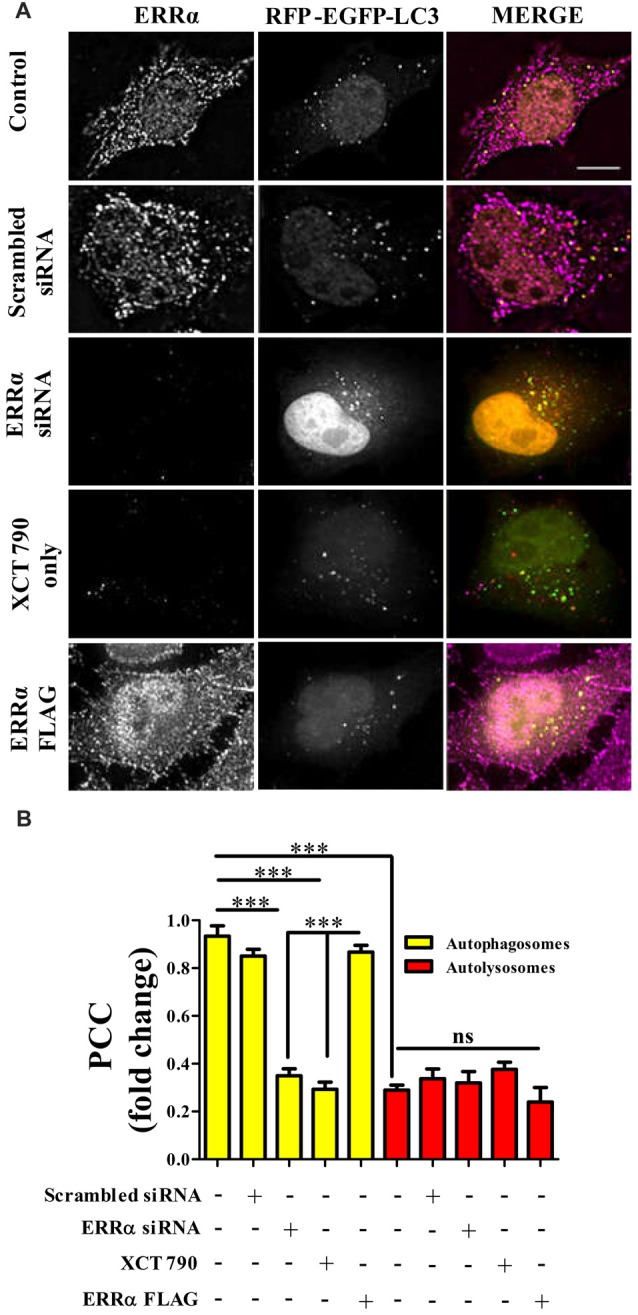
ERRα localizes onto autophagosomes to modulate autophagy. **(A)** Microscopy images of tandem RFP-EGFP-LC3 assay in HeLa cells transfected (48 h) with either ERRα siRNA or ERRα Flag treated with XCT 790 for 2 h. Cells were immunostained for ERRα. *n* = 50 cells and three independent experiments were performed. Scale bar was 15 μm. **(B)** Pearson’s colocalization coefficient (PCC) analyses of ERRα with either autophagosome (yellow) or autolysosomes (red) in HeLa cells transfected (48 h) with either ERRα siRNA or ERRα Flag treated with XCT 790 for 2 h were plotted. Statistical analysis was performed using one-way ANOVA and *post hoc* Bonferroni test. Error bars, mean ± SEM. ns-non significant, ***P < 0.001.

From knock down and over expression of ERRα studies, there was a clear indication that ERRα could modulate autophagy pathway. Autophagy was induced upon ERRα downregulation (Figures 2B,C) but inhibited when over expressed (Figures 2D,E). We examined whether active transcription was required for autophagic function of XCT 790. Upon XCT 790 treatment in the presence of actinomycin D, the autophagosomes and autolysosomes were similar to that of only XCT 790 (XCT 790 + Act D vs. XCT 790 only, *P* > 0.05, Supplementary Figures S3A,B). This result indicates that autophagic activity of XCT 790 remained unaffected when active transcription was inhibited. Subsequently, we attempted to know whether ERRα localizes to autophagic related structures such as autophagosomes and autolysosomes. Pearson’s Colocalization Coefficient (PCC) of ERRα with autophagosomes (~0.85) were found to be significantly more than that with autolysosomes (~0.3) under nutrient rich condition (~2.5-fold, autophagosomes vs. autolysosomes, *P* > 0.001, Figures 3A,B). In basal autophagy conditions, colocalization of ERRα with autophagosomes was significantly reduced in ERRα silenced and XCT 790 treated cells (~3.5-fold, untreated or scrambled siRNA vs. ERRα siRNA, *P* < 0.001, Figures 3A,B). Significantly more ERRα colocalized with autophagosomes when ERRα was over expressed (control or scrambled siRNA vs. ERRα over expressed, *P* < 0.001, Figures 3A,B). Colocalization of ERRα with autolysosomes was not regulated compared to that of control (control or scrambled siRNA vs. ERRα siRNA or XCT 790 or ERRα over expressed, *P* > 0.05, Figures 3A,B) suggesting that ERRα might not interact with the autolysosomes. ERRα could localize, most likely, to autophagosomes than autolysosomes through its non-canonical LIR motif that might interact with LC3 to localize onto autophagosomes.

These results suggest that, perhaps, ERRα might regulate autophagy through its localization with the autophagosomes.

### XCT 790 Alleviates MPTP Induced Dopaminergic Neuronal Loss

**Figure 4:**
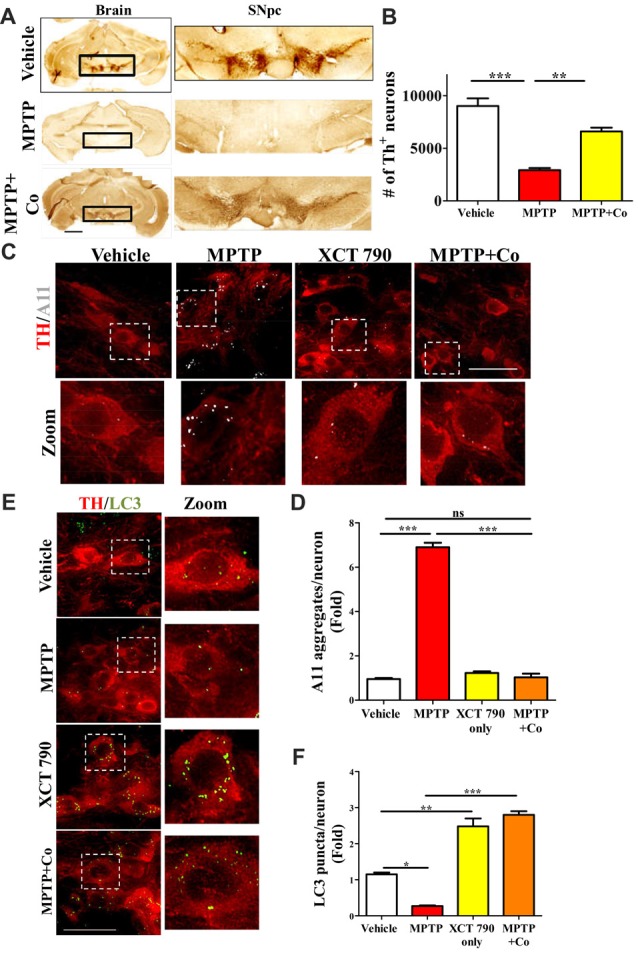
XCT 790 is neuroprotective by degrading toxic protein aggregates through inducing autophagy in dopaminergic neurons of midbrain of mice. **(A)** Representative photomicrographs of whole brain and SNpc for various cohorts namely vehicle, MPTP (23.4 mg/kg of body weight) and MPTP+Co (Co-administration of MPTP and XCT 790: MPTP; 2 mg/kg of body weight and XCT 790; 5 mg/kg of body weight, *n* = 4 animals per cohort). Scale bar is 600 μm. **(B)** Graph representing the unbiased stereological quantification of tyrosine hydroxylase (TH)-ir dopaminergic neurons in SNpc for above mentioned cohorts. Statistical analysis was performed using one-way ANOVA and the *post hoc* Bonferroni test. Error bars, mean ± SEM. **P < 0.01, ***P < 0.001. **(C)** Representative IHC photomicrographs of SNpc dopaminergic neurons double stained for A11 (toxic oligomer marker) and TH (dopaminergic marker) antibodies for the above-mentioned cohorts (*n* = 4 animals per cohort). Scale bar is 50 μm. **(D)** Plot indicating the A11 puncta per dopaminergic neuron in SNpc was quantitated for all cohorts. Statistical analysis was performed using one-way ANOVA and the *post hoc* Bonferroni test. Error bars, mean ± SEM. ns-non significant, ***P < 0.001. **(E)** Representative fluorescent IHC photomicrographs of dopaminergic neurons in SNpc double stained for LC3 (autophagy marker) and TH (SNpc marker) antibodies for various cohorts namely vehicle, MPTP, XCT only and MPTP+Co (*n* = 4 animals per cohort). Scale bar is 50 μm. **(F)** Graph representing the LC3 puncta per neuron for various cohorts. Statistical analysis was performed using one-way ANOVA and the *post hoc* Bonferroni test. Error bars, mean ± SEM. ns-non significant, *P < 0.05, **P < 0.01, ***P < 0.001.

A significant proportion of dopaminergic neurons in Substantia Nigra pars compacta (SNpc) were lost after MPTP treatment (~68%, MPTP vs. Vehicle, *P* < 0.001, Figures 4A,B, Supplementary Figure S4A) as previously described (Jackson-Lewis and Przedborski, 2007). Co-administration of XCT 790 with MPTP, however alleviated this loss by 80% (MPTP+Co vs. Vehicle, *P* < 0.05; XCT 790 vs. MPTP, *P* < 0.01, Figures 4A,B). In a congruent manner, volume of SNpc reduced significantly after MPTP injection (MPTP vs. Vehicle, *P* < 0.01, Supplementary Figure S4C), whereas the shrinkage was prevented by approximately 85% when MPTP and XCT 790 were administered together (MPTP+Co vs. MPTP, *P* < 0.01, Figure 4B, Supplementary Figure S4C).

### Cellular Tyrosine Hydroxylase (TH) Expression Was Preserved in XCT 790 Co-treatment Group

The cellular TH expression of individual TH-immunoreactive (TH-ir) dopaminergic, as measured by densitometry, was significantly reduced in surviving neurons in MPTP group (MPTP vs. Vehicle, *P* < 0.001, Supplementary Figure S4B). TH expression in the nigral neurons of MPTP and XCT 790 co-treated mice was comparable to that of the vehicle control group. Thus, XCT 790 significantly alleviated the MPTP-induced depletion of cytoplasmic TH expression (MPTP+Co vs. MPTP, *P* < 0.001, Supplementary Figure S4B).

### XCT 790 Enhances Autophagy and Clears Toxic Protein Aggregates in an *in Vivo* Mouse Model of PD

In neurons, the autophagy process is indispensable for clearing the misfolded toxic protein aggregates (Hara et al., 2006). During the neurodegenerative progression, autophagy would be defunct and becomes incompetent to maintain cellular proteostasis (Nixon, 2013). To delineate the mechanism of neuroprotective action of XCT 790, we examined the autophagy status in the various mice treatment cohorts. Our cell lines result strongly indicates that XCT 790 might exert neuroprotection through modulating autophagy. In MPTP toxicity model, the LC3 puncta per neuron was reduced significantly than that of vehicle treated (~0.8-fold, vehicle vs. MPTP treated, *P* < 0.01, Figures 4C,D) indicating the dysfunctional autophagy during neurodegenerative disease progression. Interestingly, XCT 790 only cohort exhibited significantly increased LC3 puncta per cell compared to that of vehicle treated cohort (~3-fold, vehicle vs. XCT 790 only, *P* < 0.001, Figures 4C,D). This demonstrated that XCT 790 is a strong autophagy inducer in the dopaminergic neurons of SNpc. We observed significantly increased LC3 puncta per cell in the MPTP and XCT 790 co-administered than that of vehicle treated cohort (~3-fold, vehicle vs. MPTP+Co, *P* < 0.001, Figures 4C,D). These results demonstrate that XCT 790 could induce autophagy in the SNpc of brain and remarkably surpass the autophagic deficit caused due to pathogenesis.

During protein aggregation, the toxic misfolded protein oligomeric species is shown to be accumulated in the neurons (Luk et al., 2012). We examined whether autophagy induction by XCT 790 could clear the toxic oligomeric intermediates in the neurons. In a vehicle treated cohort, the occurrences of aggregates were significantly less compared to that of MPTP treated cohort (~6.5-fold, vehicle vs. MPTP treated, *P* < 0.001, Figures 4E,F). These observations reaffirming that toxic misfolded protein aggregates are formed during disease pathology. Upon co-administration of MPTP along with XCT 790, we observed a significant reduction in the toxic aggregates compared to that of MPTP only treated cohort (~6-fold, MPTP vs. MPTP+Co, *P* < 0.001, Figures 4E,F). We found that aggregate reduction in the MPTP+Co cohort were comparable to that of vehicle treated cohort (MPTP+Co vs. vehicle, ns, *P* > 0.05. Figures 4E,F) indicating its strong potential to clear misfolded toxic protein aggregates. In addition, the presence of aggregates in the steady state level of cell in the XCT 790 only was comparable to that of vehicle cohort (vehicle vs. XCT 790 only, ns, *P* > 0.05, Figures 4E,F). This result indicates that administrated dosage regimen was not exerting any proteotoxic stress to the neurons. We demonstrated that XCT 790 could clear the pathological toxic misfolded protein aggregates upon disease progression, one of the main causative of neurodegeneration.

Mechanistically, XCT 790 exerts neuroprotection by clearing misfolded protein aggregates through inducing autophagy as demonstrated in the *in vivo* preclinical mouse model of PD.

### XCT 790 Ameliorated MPTP-Induced Behavioral Impairments

PD patients exert movement disorder symptoms such as motor co-ordination, exploration and locomotion disabilities that can be recapitulated in a MPTP mice toxicity model. As our data shows neuro-protective role of XCT 790 at both cellular and tissue level, we wished to test whether its effect can be translated up to behavioral level. To address this, we performed a set of well-known behavioral experiments—Rotarod and Open Field tests—specific for assaying the movement disorders. The scheme for behavior assay is illustrated in Supplementary Figure S5.

**Figure 5:**
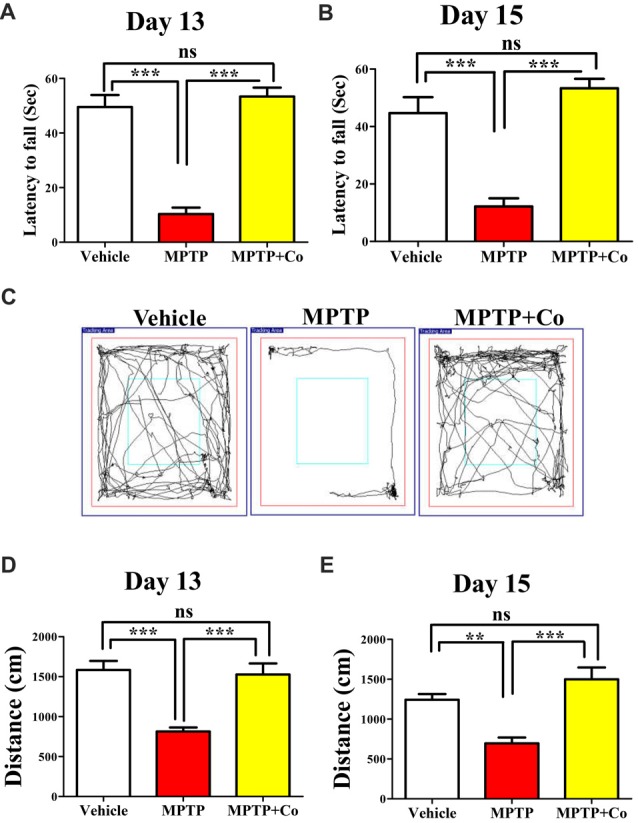
XCT 790 ameliorates MPTP-induced behavioral impairments. Latency to fall for various cohorts such as vehicle, MPTP and MPTP+Co on both day 13 **(A)** and 15 **(B)** were monitored using Rotarod test (*n* = 8 animals per cohort). Statistical analysis was performed using one-way ANOVA and the *post hoc* Bonferroni test. Error bars, mean ± SEM. ns-non significant, ***P < 0.001. **(C)** Representative trajectory maps were indicated for all the mentioned cohorts. **(D,E)** Plots indicating the peripheral distance traveled by mice were assessed through Open Field test on both day 13 **(D)** and 15 (**E**; *n* = 8 animals per cohort). Statistical analysis was performed using one-way ANOVA and the *post hoc* Bonferroni test. Error bars, mean ± SEM. ns-non significant, **P < 0.01, ***P < 0.001.

To test the exploratory ability of mice, the distance traveled in periphery zone of open field arena was compared across different cohorts. We observed that distance traveled in the zone periphery was drastically reduced in MPTP treated cohort compared to that of vehicle treated cohort (MPTP vs. vehicle control, *P* < 0.001, Figures 5D,E) on both day 13 and day 15, validating the MPTP’s effect on exploratory ability. Upon co-administration of XCT 790 along with MPTP, the distance traveled were significantly longer than that of MPTP cohort (Co vs. MPTP cohort, *P* < 0.001, Figures 5D,E), and, more importantly, comparable to that of vehicle treated cohort on both day 13 and day 15 (Co vs. vehicle control, *P* > 0.05, Figures 5D,E). Importantly, exploratory behavior of various cohorts was evident in the represented trajectory maps (Figure 5C).

Rotarod test, another standard behavioral assay to test motor co-ordination, was also employed. In this test, the time spent by mice on a horizontal rotating rod (latency to fall) was used to assess the motor co-ordination ability across different cohorts. In parallel to the results observed in Open Field, the co-treated cohort showed improved latency to fall compared to that of MPTP treated cohort (Co vs. MPTP cohort, *P* < 0.001, Figures 5A,B), which showed reduced latency to fall against vehicle treated cohort on both day 13 and day 15 (MPTP vs. vehicle control, *P* < 0.001, Figures 5A,B). In addition, the results of vehicle treated, and co-treated cohorts were fairly comparable (Co vs. vehicle control, *P* > 0.05, Figures 5A,B) on both days.

Therefore, these results demonstrate restoration of exploratory and motor coordination abilities in MPTP toxicity model, upon administration of XCT 790. Therefore, XCT 790 ameliorates the behavioral disabilities of MPTP treated mouse model.

## Discussion

Currently, there are no therapeutic interventions available for neurodegenerative diseases. New strategies to curb neurodegeneration involve identification of druggable targets, and novel small molecules are necessary. In this study, we identified small molecule XCT 790 as a novel ERRα mediated autophagy modulator that ameliorates α-synuclein toxicity and exerts neuroprotection in a preclinical mouse model of PD.

Lewy bodies are primarily due to the aggregation of misfolded proteins such as α-synuclein that exert cellular toxicity leading to neuronal death (Wakabayashi et al., 2007). Such aggregates result in perturbation of cellular homeostasis due to exaggerated proteotoxicity that trigger apoptosis and eventual loss of neurons (Hara et al., 2006). In addition, cellular proteostasis efficacy and also compensatory action among protein quality control machineries decline with age; as a result the relatively non-dividing neuronal population are more susceptible to proteostatic insult (Anglade et al., 1997). Crucial protein quality control pathways like autophagy are impaired in neurodegenerative disease pathologies (Nixon, 2013). Neurons with defective autophagy hamper the turnover of proteins and harbor protein aggregates such as ubiquitin positive inclusions and Lewy bodies (Komatsu et al., 2006). Genetic ablation of neuronal autophagy function results in progressive accumulation of neuronal aggregates and such mice manifest neurodegenerative symptoms (Hara et al., 2006). Conversely, genetically enhancing autophagy flux results in, among other things, marked clearance of autophagy adaptors (p62) that are involved in ubiquitin aggregate capture and perhaps contributing to the extended life span of such mice (Pyo et al., 2013). Corroborating such observations, pharmacological studies have implied that autophagy inducing small molecules have therapeutic potential as they restore autophagy flux which eventually mitigates neuronal loss and improves motor co-ordination in models of PD (Sarkar et al., 2007; Williams et al., 2008; Khurana and Lindquist, 2010). In this context, our study reveals the small molecule XCT 790 as an autophagy enhancer that exerts neuroprotective action.

Autophagy, a tightly regulated process is maintained at low (basal) levels during fed condition and elevated in presence of stress such as starvation (Hara et al., 2006). This low level of autophagy is maintained by negative regulation by MTOR. We now show that, in addition to MTOR pathway, ERRα negatively regulates autophagy flux. When the ERRα inverse agonist, XCT 790, relieves this regulation, the number of autophagosomes and autolysosomes increase several folds. Furthermore, it is known that MTOR activity is not perturbed in the absence of ERRα (Chaveroux et al., 2013). We, therefore, investigated the interdependance of MTOR and ERRα in controlling autophagy and show that XCT 790 induces autophagy independent of MTOR signaling, a well-desired characteristic for further drug development to combat adverse side effects. In addition, MTOR regulates transcription of Ubiquitin Proteasome System (UPS) related genes leading to degradation of ERRα upon induction of autophagy by rapamycin (Chaveroux et al., 2013). Our results suggest that autophagy inhibiting activity of ERRα in nutrient rich conditions is through MTOR independent mechanism. In autophagy triggering conditions, where basal inhibitory activity needs to be removed, ERRα gets ubiquitinated and degraded by UPS (Chaveroux et al., 2013), that corroborates our findings.

Among the MTOR independent pathways, AMPK regulates autophagy flux. Previously, XCT 790 was reported to modulate AMPK pathway in ERRα independent fashion (Eskiocak et al., 2014). We observed XCT 790 modulates autophagy by not regulating the AMPK pathway, one of the reported MTOR independent pathways, but through subcellular ERRα localization dynamics.

XCT 790 is reported to be the most selective inverse agonist of ERRα (Eskiocak et al., 2014). Though it is known that ERRα localizes primarily in the cytoplasm (Sladek et al., 1997; Ju et al., 2009), its cytoplasmic function is not yet reported. Thus, apart from its transcriptional function in nucleus (Sladek et al., 1997), our findings reveal a cytoplasmic role for ERRα in autophagy. Recently, ERRα has been shown to up regulate autophagy related genes in the context of clearance of *Mycobacterium* in a transcriptional-dependent manner (Kim et al., 2018). Our results further demonstrate that XCT 790 not only induces aggrephagy but also clears intracellular *Salmonella* burden via recruiting autophagy related proteins such as LC3 and p62. Thus, ERRα uniquely induces selective autophagy pathways by both transcriptional-dependent and -independent mechanism to clear misfolded aggregates and intracellular pathogens.

## Author Contributions

RM, SNS and AKC conceived the project. SNS, MP, AKC, DJV and HY carried out the experiments. PAA supervised the mice histology experiments. JPC and RM designed the behavior experiments. SR, AP and SNS performed the behavior experiments. SNS, JPC and RM analyzed the behavior results. VA conducted the xenophagy experiments. RM, SNS, MP, DJV, HY, JPC, SR and PAA analyzed the results and wrote the manuscript. All authors approved the manuscript.

## Conflict of Interest Statement

The authors declare that the research was conducted in the absence of any commercial or financial relationships that could be construed as a potential conflict of interest.
