# Supplementary material for: Modulation of Autophagy by a Small Molecule Inverse Agonist of ERRα Is Neuroprotective
Source: Front Mol Neurosci. 2018 Apr 9;11:109. doi: 10.3389/fnmol.2018.00109 (PMC5900053; doi:10.3389/fnmol.2018.00109)
Supplement: Supplementary file 1 [file Data_Sheet_1.docx]

**Supplementary figure legends**

**Figure S1: XCT is non-toxic to cells (HeLa and SH-SY5Y) and scheme for α-synuclein toxicity assay.**

Cell viability of cell lines like HeLa (A) and SH-SY5Y (B) after 72 h of XCT 790 treatments for various indicated concentrations. Cell viability was assayed using CellTitre Glo (Promega™) kit (n=4 and three independent experiments).

**Figure S2: XCT 790 modulates autophagy in MTOR-independent manner in SH-SY5Y cells.**

(A) Representative microscopy images of tandem RFP-EGFP-LC3 assay in SH-SY5Y cells treated with XCT 790 for 2 h. Yellow puncta were autophagosomes and red were autolysosomes. Fold change in autophagosomes and autolysosomes by XCT 790 were quantified and plotted (n=50 cells, three independent experiments). Scale bar was 15 μm. Statistical analysis was performed using two-tailed paired t-test. Error bars, mean ± SEM. *-P < 0.05, **-P < 0.01.

(B) Representative Western blots of MTOR substrates like P70S6K (phospho and total form) and 4EBP1 (phospho and total form) regulation by various treatments like XCT 790, EBSS and LiCl in SH-SY5Y cells (n=4 and three independent experiments). β-tubulin was used as a loading control.

**Figure S3: Autophagic function of XCT 790 was unaffected in presence of actinomycin D.**

(A) Representative microscopy images of tandem RFP-EGFP-LC3 assay in HeLa cells co-treated with XCT 790 and actinomycin D (act D). Scale bar 15 μm.

(B) Fold change of autophagosomes and autolysosomes across various treatments were plotted (n=50 cells, four independent experiments). Statistical analysis was performed using one-way ANOVA and post-hoc Bonferroni test. Error bars, mean ± SEM. ns-non significant.

**Figure S4: Administration of XCT 790 in mice MPTP-toxicity model.**

(A) Dosage regimen of XCT 790 in various cohorts namely vehicle, MPTP (23.4 mg/kg of body weight) and MPTP+Co (MPTP; 23.4 mg/kg of body weight and XCT 790; 5 mg/kg of body weight).

(B) Plot indicating the densitometric quantification (B), measure of TH intensity in dopaminergic neurons (n=4 animals per cohort). Statistical analysis was performed using one-way ANOVA and post-hoc Bonferroni test. Error bars, mean ± SEM. ***-P <0.001.

(C) Plot indicating the nigral volume for the cohorts. (n=4 animals per cohort). Statistical analysis was performed using one-way ANOVA and post-hoc Bonferroni test. Error bars, mean ± SEM. **-P <0.01, ***-P <0.001.

**Figure S5: Scheme for the behavior study.**

Scheme indicating the dosage regimen of various cohorts such as vehicle (A), MPTP (B) and MPTP+Co (C) followed for the behavioral study.

**A**

**B**

**Figure S2**

**Figure S3**
